# Women’s Postpartum Experiences of Hypertensive Disorders of Pregnancy: A Qualitative Study of Barriers and Enablers to Healthy Lifestyle Behaviours

**DOI:** 10.3390/ijerph23010100

**Published:** 2026-01-11

**Authors:** Lynne Roberts, Chris Rossiter, Elizabeth Denney-Wilson, Megan Gow, Amanda Henry

**Affiliations:** 1 Women’s and Children’s Health St George Hospital, Sydney 2217, Australia; ahenry@georgeinstitute.org.au; 2 St George and Sutherland Clinical Campus, School of Clinical Medicine, University of NSW Medicine and Health, Sydney 2217, Australia; 3 Susan Wakil School of Nursing and Midwifery, Faculty of Medicine and Health, The University of Sydney, Sydney 2006, Australia; christine.rossiter@uts.edu.au (C.R.); elizabeth.denney-wilson@sydney.edu.au (E.D.-W.); 4 Sydney Local Health District, Royal Prince Alfred Hospital, Camperdown, Sydney 2050, Australia; 5 The George Institute for Global Health, UNSW Medicine and Health, Sydney 2031, Australia; mgow@georgeinstitute.org.au; 6 School of Population Health, Faculty of Medicine and Health, The University of New South Wales, Sydney 2052, Australia; 7 Discipline of Women’s Health, School of Clinical Medicine, Faculty of Medicine and Health, The University of New South Wales, Sydney 2052, Australia

**Keywords:** hypertensive disorders of pregnancy, heart disease risk factors, health behaviour, healthy lifestyle, qualitative research, postpartum health

## Abstract

**Highlights:**

**Public health relevance—How does this work relate to a public health issue?**

- HDP markedly increase long-term cardiovascular risk, making postpartum lifestyle support a key public health priority.
- Women’s experiences reveal behavioural and system-level factors influencing their ability to adopt healthy behaviours.

**Public health significance—Why is this work of significance to public health?**

- The study identifies modifiable barriers to recommended lifestyle behaviours during a critical prevention window.
- Findings highlight enablers that can inform targeted, woman-centred interventions.

**Public health implications—What are the key implications or messages for practitioners, policy makers and/or researchers in public health?**

- Clinicians should provide clear risk communication, tailored guidance, and coordinated postpartum follow-up.
- Policies and research should support accessible, culturally appropriate resources to reduce long-term cardiovascular risk.

**Abstract:**

*Background:* Hypertensive disorders of pregnancy (HDP) have significant implications for women’s long-term health, including at least a twofold increased lifetime risk of cardiovascular disease (CVD). The Blood Pressure Postpartum (BP^2^) Study was a three-arm randomised trial evaluating follow-up and lifestyle behaviour change strategies during the first year after HDP. *Methods:* This qualitative sub-study, embedded within the BP^2^ Study, explored women’s experiences of life in the first year following HDP. Semi-structured telephone interviews were conducted with 34 women, approximately 10–12 months postpartum. Interviews were transcribed verbatim and a thematic analysis was undertaken. *Results:* Participants reflected on their experiences post-HDP; three major themes were identified: *Navigating life with a newborn*, *The value of support*, and *Processing and Moving forward*. Some women felt informed and empowered to make positive lifestyle changes; others were still processing their HDP experience and/or feeling overwhelmed by the demands of early motherhood. Responses were influenced by their HDP experience, available support, prior experience with healthy behaviours, and financial stability. *Conclusions:* The findings highlight that postpartum women who experienced HDP face unique challenges, including physical recovery, emotional processing, and intensive infant care. It often takes time for these women to begin prioritising their own health, as they navigate these challenges. The insights generated from women’s experiences suggest that flexible, accessible, and individually tailored support may facilitate postpartum health, promote lifestyle change, and help reduce long-term CVD risk.

## 1. Introduction

Hypertensive disorders of pregnancy (HDP) complicate 3–8% of pregnancies globally and are a leading cause of maternal and perinatal morbidity and mortality [1]. HDP include chronic hypertension (CH): hypertension existing prior to pregnancy or diagnosed in the first 20 weeks of pregnancy; gestational hypertension (GH): new-onset hypertension after 20 weeks’ gestation; and preeclampsia: new-onset hypertension after 20 weeks’ gestation accompanied by maternal and/or fetal end-organ dysfunction [2,3]. HDP can lead to a range of complications for the mother and the baby. Consequently, women diagnosed with HDP may require specialised care, increased pregnancy surveillance and/or antenatal hospital admissions, and a longer postpartum stay in hospital, and they commonly give birth pre-term. Furthermore, the risks of HDP extend beyond the pregnancy and immediate postpartum period, with several meta-analyses demonstrating that women have a 2–4-fold increased risk of future cardiovascular disease (CVD) following HDP [4,5,6,7]. This increased risk may become evident within the first five years post-HDP and continue lifelong [8,9].

CVD is an umbrella term that includes heart disease, stroke and blood vessel disease [10]. It is the leading cause of illness and death worldwide [11], contributing to 35% of female deaths [12]. There is increasing recognition that many incidences of CVD can be prevented through healthy lifestyle behaviours. Preventing CVD is a public health priority that requires action and, due to their increased risk, is imperative for women who have experienced a hypertensive pregnancy.

International guidelines [2,3] recommend that women should be advised of their increased long-term health risks prior to their post-HDP hospital discharge and be made aware of ways to mitigate risk. Evidence suggests that behaviours such as healthy diet, regular physical activity, weight management, smoking cessation, and reduced alcohol consumption, can reduce the risk of CVD [13]. However, the postpartum period and transition into parenthood are difficult times, and it may be challenging to adopt and adhere to a healthy lifestyle [14]. Furthermore, a change in lifestyle behaviour may be more difficult for women who are processing the stress related to a pregnancy complicated by hypertension and/or preterm birth [15].

The Blood Pressure Postpartum (BP^2^) Study was a multicentre three-arm randomised control trial (RCT) evaluating lifestyle behaviour change strategies in the first year after birth to reduce long-term cardiovascular risk following HDP. This qualitative sub-study, embedded within the BP^2^ Study, explored women’s experiences in the first year post-HDP and the barriers and enablers to adopting healthy lifestyle behaviours following a hypertensive pregnancy.

There is limited qualitative evidence capturing women’s lived experiences during the first year post-HDP, including perceived barriers and facilitators to adopting healthy behaviours. This study addresses this gap by providing in-depth insights into how women navigate recovery, caregiving, and lifestyle change following HDP, offering novel perspectives that can inform the design of tailored postpartum interventions and long-term CVD prevention strategies.

## 2. Materials and Methods

### 2.1. The Blood Pressure Postpartum Study

Women diagnosed with HDP were recruited within six months postpartum from five Sydney hospitals, which collectively oversee 19,000 births annually and serve a socio-demographically diverse population from the Sydney metropolitan area and regions further afield. Eligible women were approached by research midwives, received study information, and were enrolled upon consent.

At six months postpartum, women completed online lifestyle/health surveys prior to randomisation into one of three groups:

- Group 1. Optimised Usual Care—information brochures given and GP review.
- Group 2. Brief Education Intervention—information brochures given, anthropometric measures taken, plus a dietitian and physician consultation.
- Group 3. Extended Lifestyle Intervention—the Brief Education Intervention plus referral to the NSW Get Healthy Service (six-month telephone lifestyle coaching service from a qualified dietitian or exercise physiologist).

All participating women attended their study site at 12 months postpartum for follow-up measurements. The primary outcomes were change in systolic blood pressure, and weight and waist circumference from six months to 12 months postpartum. The detailed study protocol has been previously published [16].

### 2.2. Ethics

The study was approved by a local health district human research ethics committee (2019/ETH04732). BP^2^ was prospectively registered with ANZCTR as a clinical trial (ACTRN12618002004246).

### 2.3. Research Design

This sub-study used semi-structured interviews with some BP^2^ study participants. It addressed the research question: What are women’s experiences post-HDP and how does this affect adopting healthy behaviours? The interviews were also used to address the research question of the impact of the study interventions on women’s health behaviour following HDP, and these findings have been previously published [14].

### 2.4. Recruitment

In the six-month postpartum online BP^2^ survey, participants were invited to consider the optional telephone interview for this sub-study. Those who expressed interest received further information and the consent form. The BP^2^ Project Manager (LR) obtained informed consent then released participant contact details to the interviewer (CR). The interviewer contacted each woman and arranged a mutually convenient time to conduct the interview. The researchers recruited purposefully to ensure a similar number of women from each intervention group were interviewed. Women received a gift voucher for $30AUD as remuneration for their time.

### 2.5. Interviews

At 10–12 months postpartum, the semi-structured interview was undertaken via telephone and audio-recorded. Interviews were conducted between March 2020 and April 2021 until data saturation was reached, defined as no new or divergent themes emerging, ensuring comprehensive coverage of participants’ experiences. Some interviews were conducted during the COVID-19 lockdowns in Sydney. While the lockdowns had a substantial impact on women’s ability to make lifestyle changes, this issue is not explored in detail here as it has been reported previously [17].

The interview schedule was developed by the BP^2^ research team, including a consumer representative, and guided by the Social Ecological Model [18], which recognises that behaviour is shaped by interconnected personal and environmental influences. Questions focused on diet, eating habits, physical activity, and other health behaviours, with the aim of identifying barriers and facilitators to healthy lifestyle practices across individual (knowledge, attitudes, behaviours), interpersonal, community, and societal levels (see Appendix B for interview guide). The interview schedule was pilot-tested and refined by the interviewer (CR), a female social scientist experienced in qualitative research and independent of other aspects of the BP^2^ Study. Reflexivity was addressed through awareness of the interviewer’s disciplinary background and by maintaining field notes to document contextual observations and analytic reflections following each interview. The interviewer conducted the telephone interviews using a series of open-ended questions, which were applied flexibly to maintain conversational flow and to allow women to share their experiences.

### 2.6. Data Analysis

After the interviews were completed, audio recordings were de-identified, professionally transcribed, and checked for accuracy. Participants were invited to review their transcripts for validation, but no one chose to do so. Transcripts were imported into NVivo qualitative data analysis software (Version 12; QSR International) for data management. Data were analysed using the thematic analysis approach outlined by Braun and Clarke [19], consisting of familiarisation with the transcript data; coding the dataset in relation to the research question; identifying themes across the coded data; reviewing, defining and naming the themes; and finalising the analysis. This approach allowed patterns and themes to emerge from the interview transcripts. The interviewer (CR) and authors (LR, EDW and AH) reviewed the interview transcripts and performed the analyses. These four authors (LR, AH, CR, EDW) were also involved in discussing and developing codes, themes, and sub-themes until agreement was reached. Direct quotes are provided to illustrate the themes. To protect the woman’s identity, a code (interview number (#), parity (primipara or multipara) and study Group allocation), is included at the end of each quote.

## 3. Results

A total of 34 BP^2^ participants were interviewed: 12 from Group 1, and 11 each from Groups 2 and 3. The interviews ranged from 18 to 47 min in length. Interviewees were comparable in maternal age, education level and gestational age at giving birth. Compared to the BP^2^ cohort overall, a higher proportion of interviewees were born in Australia, and had only one child at the time of the interview. Table A1 summarises demographic characteristics of the interviewees compared with the whole BP^2^ cohort.

While this qualitative sub-study did not aim to compare experiences across the three BP^2^ intervention groups, it is possible that differences in intervention exposure may have influenced women’s perceptions of support, motivation, or opportunities for lifestyle change. The findings therefore reflect participants’ reported experiences within the context of the intervention they received but were not analysed comparatively in this manuscript.

### 3.1. Themes

**Figure 1:**
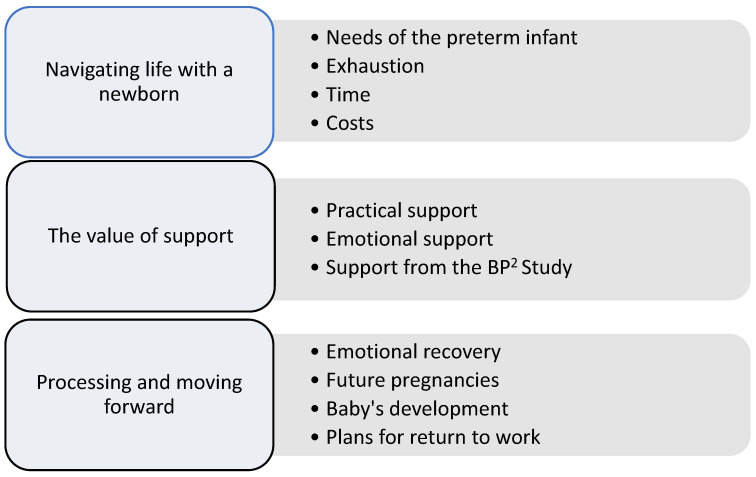
Themes and sub-themes derived from thematic analysis of interview transcripts in the BP^2^ qualitative sub-study. Themes represent interconnected aspects of women’s post-HDP experiences and are presented conceptually. BP^2^; Blood Pressure Postpartum Study.

Three main themes were identified, reflecting women’s post-HDP experiences and their influence on adopting healthy behaviours: *Navigating life with a newborn*, *The value of support*, and *Processing and Moving forward*. Each theme was further supported by associated sub-themes shown in Figure 1.

#### 3.1.1. THEME 1: Navigating Life with a Newborn

Most respondents recounted the significant impact of having a young baby in their lives, including their capacity to engage in healthy lifestyles. This was particularly true for the first-time mothers. Several described feeling overwhelmed by the constant demands on them:

*“For me, the biggest challenge is just the childcare aspect of it. And the broader time within your day, just to run your life.”*

#08, primipara, G1.

*“I’m just, I think, in this fight or flight mode right now, it’s just like, keep the baby alive.”*

#22, primipara, G2.

For women who experienced a premature birth, the transition to parenthood was further complicated by the specific needs of their preterm baby and ongoing concerns about their child’s health. Many mothers described delaying attention to their own recovery and wellbeing to prioritise their baby’s care.

*“Our main challenges would probably be, for me, I find because everything’s so busy with maintaining the home, with making sure that [baby] attends to all her appointments. I’m not sure if you’re aware she was premature…. she’s got a slight delay in her gross motor…So, we’re seeing a physio every three weeks…with all the appointments and all the things that are happening, Sometimes it can get a little bit tiring.”*

#31, primipara, G3.

*“I have exercised before. So, I feel like I think it was just something that I had to put on hold during [baby]’s pregnancy and for a fair few months afterwards. I guess, the reason of having a caesarean, but also because she was so little and she was unwell. I think it probably was something that I had in me; I just had to pause it.”*

#32, multipara, G3.

Several mothers discussed the lack of sleep and its impact on their levels of energy. They pointed to exhaustion and their inability to focus or be organised.

*“I’m not that focused on food as much as I used to be, and because I’m tired by the end of the day, often I don’t really care what we eat.”*

#07, primipara, G2.

*“Making healthy choices is more difficult when you’re extremely sleep deprived”*

#30, primipara, G3.

Most women highlighted that adjusting to parenting meant having less time available. They compared this to their life before motherhood when they had fewer constraints on their capacity to exercise or make time for themselves. Many respondents discussed having limited time and opportunity for physical activity now that they had young babies:

*“I bought a running pram to try and get back into it and stuff. It’s just been too hard because she doesn’t sleep in the pram…I suppose if I really tried, I could get back into it, but I’ve found it quite problematic getting back into running.”*

#22, primipara, G2.

*“I used to play hockey and tennis and those sorts of things. It would be great to be able to do that again, but again, the timing of fitting it in and the cost involved…. We’d have to sort of take turns, I guess. One of us goes out to do sport one evening and the other looks after the kids. Unfortunately, at the moment it’s low on the priorities.”*

#24, multipara, G1.

The demands of parenthood also affected women’s ability to eat healthily. They often reported not having time to prepare nutritious meals, especially as they were now preparing solid food for their baby in addition to other family meals. Some reported having little time to eat.

*“Sometimes I’m that busy I don’t eat anything until dinner.”*

#15 primipara, G1.

*“Very quickly in the morning I will feed her something and I will just have a yoghurt or something like that, quickly. I don’t do anything about my food right now, it’s more about baby’s food.”*

#22, primipara, G3.

Several women noted that they resorted to choosing convenient options or snacking unwisely to maintain their energy. They mentioned “comfort eating”, “grabbing the easy things” or “just getting through the day”. Others noted:

*“I definitely could be incorporating more vegetables into my diet. I guess I just, at this point, with a small baby, I guess I’m just preferencing convenience.”*

#30, primipara, G3.

*“I think especially nowadays with Deliveroo and everything just being so handy, it’s a lot easier to just sit on my phone and order something…. I’d much rather do a takeaway shop [rather than] doing that shopping, and it’s actually buying it and then preparing it, and finding the time to actually prepare it. So, I’d say they’re the biggest challenges.”*

#32, multipara, G3.

Several women discussed having little or no childcare, limiting their ability to engage in team sports or sessions at a gym or Pilates studio. Others discussed the difficulty of affording gyms or healthy food options which are typically more costly than alternatives.

*“I used to go to the gym quite regularly. When I had [baby], I just didn’t have the opportunity, because I don’t have family here. They’re all abroad.”*

#02, primipara, G3.

*“Good food, I found the other day, is really more expensive than junk food… I can see why people do go and form that unhealthy lifestyle very quickly, because they can’t afford to eat—it’s terrible.”*

#18, primipara, G2.

*“I know there’s a lot of boot camps, they have for like mums and bubs type of thing, but…my money’s running out soon. My maternity leave pay is finished. My partner, he’s covering everything. So, it’s hard to try and get out extra money to try and do stuff.”*

#19, multipara G3.

Overall, many respondents highlighted the complexity of trying to maintain good health in the early days of parenthood. One mother summed up her frustration:

*“It adds just the pressure of everything else, adjusting to parenthood, and getting through the day, and on top of that, oh, no, regardless how exhausted you are, you have to go and keep up your activity levels. No, you can’t go and get a convenience meal. You can’t go and get takeaway. You have to go and cook from scratch because you need to make sure you have a low sodium diet. It just adds a lot.”*

#34, primipara, G3.

#### 3.1.2. THEME 2: The Value of Support

The interviews highlighted the importance of practical support to help mothers keep healthy when they have a new baby. They often mentioned help with childcare to enable them to exercise, and support with preparing nutritious meals.

*“My husband has been really, really supportive. When I was trying to work out at home, he takes the baby, so that I could get my one hour, fit in a quick exercise. But, I don’t really have family around. My family is back in [country].”*

#09, primipara, G2.

*“My mum’s very healthy, so that’s been good too. She’s always got good food ready for me to take home, to bring over…. she’s [also] very supportive of me staying fit.”*

#21, primipara, G3.

*“My friends are really good. There was five of us that had babies within three months of each other. And a couple of the girls are incredibly sporty, which I think has been a good influence on me. And they introduced me to these classes actually, which I might not have tried having not got the advice from them…. And then also, my husband is great and supportive of it.”*

#28, primipara, G1.

Mothers also highlighted the importance of more emotional support from others, through encouragement, understanding or a sense of solidarity and shared purpose:

*“We cook at home a lot. So nutritionally, I would say it’s much better… It’s also something that my husband and I like to do together. So, that’s a benefit.”*

#03, primipara, G2.

*“My Mum is normally pretty good. Whatever I try she normally tries with me and her work was doing a weight loss sort of competition… So we’re both trying to eat healthy and not eat so much crap really. That helped having somebody else to sort of talk to about it.”*

#13, primipara, G2.

Although much support came from partners, family and friends, some mothers also reported feeling supported by broader communities especially mothers’ groups.

*“Like we’ll put them on the floor or like you can have them still in their pram or whatever [while we exercise]… it was good that someone, someone in the mother’s group like found the class and suggested the class and everyone sort of got on board.”*

#01, primipara, G1.

*“They [mothers’ group] often come for a walk with me…I was the driving force behind being a little more active and doing that. But yeah, a couple of them jumped on board.”*

#06, primipara, G1.

The mothers also mentioned the support they received from the BP^2^ study team and, for Group 3, the Get Healthy Service (GHS). They valued the attention and regular, understanding feedback from the GHS coaches, personalised to their own needs and circumstances. The follow-up phone calls provided accountability and an incentive to maintain healthy regimes.

*“… I’m not sure if this BP^2^ intervention is available to all mums, so I definitely feel that this research, or these programs that are out there, definitely will assist. I guess it’s just because I feel as a first-time mum there’s so much to think about. … otherwise I feel that I would have just fallen into a hole and then left myself there.”*

#31, primipara, G3.

*“The Get Healthy Service is great I think. It just meant that I had someone guiding me a little bit better and I didn’t tend to get lazy, because I knew that someone would be calling to ask how I’m doing and I’m a terrible liar, so I kept it up.”*

#21, primipara G3.

Conversely, several of the mothers attributed their difficulties maintaining healthy behaviour to limited support. This exacerbated the pressures of new parenthood. Several mothers explained that their family lived overseas and other family members were not always supportive or helpful.

*“My family live in [country] and my husband’s are in [country]. So, we have no family support, really, for that time if I want them to look after [baby] while I went, and things. So that [regular exercise] did drop off.”*

#02, primipara, G3.

*“My husband…likes to exercise and he likes to play football…. it’s now annoying because if he comes home and so little time that he can spend with the baby and then it’s like, ‘Oh, I have a soccer game’…I’m like, ‘What? You should spend time with the baby and not play soccer.’*

#22, primipara, G2.

Several mothers reported frustration at actions that worked against their intentions to achieve better health.

*“We have a gym at work that is free for everyone to use…during work time…But it just depends who your line manager is, whether or not they support you doing that during work time… unfortunately my boss does not support that.”*

#06, primipara, G1.

*“[My partner] is the one that if he goes off to get milk and bread, he comes home with a block of chocolate as well every now and then. If it’s not there, I don’t eat it. If it’s just there, you eat it.”*

#14, primipara, G1.

*“I don’t want to throw him under the bus, but sometimes my partner will take the easy way out and just get Maccas or pizza and I really try hard to be balanced.”*

#21, primipara, G3.

#### 3.1.3. THEME 3: Processing and Moving Forward

The HDP and birth experience was often described as traumatic, with effects that persisted for many months postpartum. As mothers worked to process their experiences, attention to their own health was frequently delayed:

*“I had a very traumatic birth and a very traumatic pregnancy…I spent a lot of time trying to process it and I think I’ve just processed it all only recently and started to feel a bit better about myself.”*

#13, primipara, G2.

Many women spoke openly about the emotional impact of their HDP experience, describing how it affected their mental health. They reflected on the stress, anxiety, and uncertainty that accompanied early motherhood, and emphasised that exercise provided a crucial way to cope with these feelings. For many, physical activity was more than just a means of staying fit, it offered a sense of control, a space to process emotions, and a way to support their overall recovery and wellbeing:

*“My health, like I had postnatal depression and hypertension, so I was in hospital longer……. being able to get back and do even 10 min [exercise] a day for the mental health benefits of that was really, really important. So, it’s good now to be able to do more.”*

#05, multipara, G3.

*“For me it was my mental health as well, I started seeing a therapist just once every two weeks or once a month just to talk about how it’s like being a new mum of twins…there’s a lot of things that can be quite challenging, but I feel as though, if you’re across your mental health and you have a lot of support around you, then you’ll be fine.”*

#21, primipara, G3.

*“I go through stages, and I think when my mental health changes a bit, that’s when I just keep going down a slide. Then when I kind of get on top of that again, I’m like, ‘Oh no, I can actually do this’. So, it’s just trying to find a consistent level where…I can still maintain a healthy lifestyle and diet.”*

#25, primipara, G3.

Most interviewees discussed how they might focus on their health in the future, and what factors would influence their adoption and maintenance of healthy behaviours. Several women addressed their future health and the potential recurrence of HDP in future pregnancies as motivating factors. Some mentioned that they planned to focus more on keeping healthy in their next pregnancy or being better informed, often because of what they had learned through the BP^2^ study.

*“Because I had preeclampsia and I know there’s a risk of it happening again in another pregnancy, I want to try to… I feel like if I can be healthier for the next pregnancy, then that will benefit me. It may or may not help with preeclampsia, but just being more prepared, I guess… better informed and physically and just that, I’ve kind of put myself in the best position.”*

#07, primipara, G2.

*“So, I always try to make sure if my blood pressure isn’t great. I always try to make sure that I’m eating properly to suit that as well as trying to exercise to see if that’ll help. Just get it down that little bit.”*

#06, primipara, G1.

*“I’m now following the DASH diet as best as I can… It’s basically a diet that is meant to help reduce risk factors for high blood pressure…There is that motivational thing in the background that if I don’t make this a habit and if I don’t try to maintain this, then I may have these complications that I don’t want to develop later on in life.”*

#34, primipara, G3.

Many of the women discussed factors which would help them maintain good health in the future. Several noted that preparing healthy food was easier now that their babies were older and starting to share meals with the rest of the family.

*“She [dietician] was like, ‘Yeah, if you’ve got a healthy diet, you can just feed her what you’re eating’, which means you’re just making one meal. I make a meal in the evening, and then I keep some of it for the next day for [baby] to have. And it’s not fussy. It makes it really easy. You hear of people and they’re doing all these steamed vegetables, and making all this stuff. And it’s like, ‘Oh, when am I going to have time to do that?’ But you actually don’t have to. If you’re eating healthy, …. I can give that to [baby] as well.”*

#02, primipara, G3.

*“I feel like now that [baby] is eating solids, it’s a bit easier because I want to be a good example for her, and I want us to try and eat the same, like similar things. But in that first six-month period, making that priority was really hard.”*

#29, primipara, G2.

Some mothers highlighted that as their children were developing and becoming more settled into routines, it was possible to focus on keeping themselves fit or to enjoy physical activity as a family.

*“I am really trying to get healthy and that’s helped as well like taking more time to focus on myself now that my daughter’s…got a routine now. I know what time she goes to bed. I know what time she naps so I can sort of fit in a workout or I can just do like wash my hair. Just sort of do something for myself.”*

#13, primipara, G2.

*“At the beginning it was a bit harder, I think. She just preferred to be with me, but now she’s 10 months now, she’d be more than happy for an hour to go to the park with him [partner]. So, it feels like it’s getting a lot easier.”*

#16, primipara, G1.

Interviewing mothers at approximately 10 months postpartum meant that many were returning to paid work and planning to leave their infants with relatives or in childcare. For some the return to work provided a more predictable routine and the opportunity to have a little more time to focus on their own health.

*“I think when I do go back to work that in some ways that’ll get me a bit more structure as well, I’ll be a bit more focused. And I’ll have a bit more adult contact which will be a bit more stimulating.”*

#08, primipara, G1.

*“Probably going back to work might make things a little bit easier too. So, [baby] will be with my mum or my mother-in-law and I could have some time to myself when I get home from work. I know that they would jump at the opportunity to have an extra hour if I decide to go to the gym after work.”*

#14, primipara, G1.

*“But I’ve gone back to work now, so I guess my diet, I actually had more time to be a little bit more structured… because I have the time to be able to eat at work… Being back at work, because of what I do, it’s a little bit more physical. So that keeps me moving as well.”*

#18, primipara, G2.

Other mothers were concerned about the impact of returning to paid work on their ability to keep healthy and active given the conflicting demands of home and work, especially for those in demanding jobs.

*“I feel if I’m struggling to fit it in and prioritise it and make it work in my day-to-day life now, that could be a challenge when I go back to work… I really want to make it; surely I can prioritise me for one hour a week. But the actual logistics of making that happen is a bit challenging.”*

#04, primipara, G3.

*“I think when I go back to work, the bigger challenge will be adding that complication. How do I do everything to keep the house going and to keep him healthy and happy—and work?”*

#08, primipara, G1.

## 4. Discussion

This qualitative study provides insight into women’s lived experiences following a pregnancy complicated by HDP. It highlights how the emotional and physical impacts of HDP, and in some cases, the challenges of caring for a preterm infant, added layers of challenge to the postpartum period and shaped their ability to engage in postpartum lifestyle change. These qualitative insights can inform long-term CVD prevention strategies by highlighting the types of support and interventions that may enhance sustained lifestyle change.

Many women described needing time to process their HDP experience before they could focus on their own health, reflecting the highly individualised nature of readiness for postpartum interventions. While structured follow-up is increasingly recommended to mitigate future CVD risk, our findings demonstrate that women’s readiness to engage with such interventions is highly variable.

The demands of infant care emerged as a key barrier to postpartum lifestyle change. Women caring for preterm or health compromised babies (a common consequence of HDP) reported frequent medical appointments, heightened concern for their child’s health, and intensive caregiving, alongside sleep deprivation and breastfeeding challenges. Many described delaying attention to their own recovery while prioritising their baby’s needs. This finding aligns with prior research indicating that postpartum women face substantial challenges to engaging in healthy behaviours due to competing demands [20]. However, our findings and others [21] also suggest that as routines stabilise over time, women become more able to focus on their own wellbeing. This points to the need for lifestyle interventions that are flexibly timed and adapted to women’s evolving readiness during the first postpartum year.

Support from family, friends, and healthcare professionals was identified as a critical enabler of lifestyle change. Women with dedicated support networks reported greater success in engaging in health-promoting behaviours, whereas those with limited support described more difficulty. This is supported by existing research showing that support from partners and extended families facilitates regular physical activity and healthy eating [20], and that social support increases motivation and engagement in health behaviours over the first two years postpartum [22]. In contrast, limited perceived support is linked to reduced attendance at postpartum care visits [23], which are key opportunities for health promotion.

These findings underscore the value of integrated care models that embed psychosocial support within lifestyle interventions. Strategies such as involving family members in education sessions, offering peer support groups, or providing outreach via community health workers may be effective. Addressing broader structural barriers, including financial stress and childcare access, can also improve women’s capacity to engage in healthier behaviours.

As the postpartum period progressed, many women described a growing readiness to prioritise their health. This shift was often facilitated by processing their HDP experience, reduced caregiving demands, greater awareness of long-term risks, and the desire to improve health for future pregnancies, to optimise maternal and fetal outcomes, and overall wellbeing. HDP is well recognised as potentially traumatic [15,24], and women may require many months to process and make sense of their pregnancy and birth experience [15]. In addition, HDP can contribute to or exacerbate postpartum mental health disorders [25], which may further influence women’s readiness to adjust to parenthood and engage in health-promoting behaviours. Several participants attributed their increased motivation to improve their lifestyle and health to the information received during the BP^2^ study. As children became more settled into routines, women found it easier to prepare healthy meals and incorporate physical activity, sometimes as a family. These experiences align with earlier findings that maternal readiness for lifestyle change improves over time as daily routines become more manageable [20,26]. Returning to paid work introduced a mix of benefits and challenges, offering structure and time for self-care for some, while adding stress for others, reinforcing the need for interventions that are responsive to individual life contexts.

Overall, the findings highlight the need for postpartum care that is flexible, accessible, and tailored to women’s readiness and individual circumstances following HDP. In practice, this could be supported through structured post-HDP pathways that integrate primary care follow-up, routine monitoring of cardiovascular risk factors, and referrals to community-based lifestyle programmes such as diet, physical activity, or mental health support. Embedding these supports within existing postpartum care frameworks may help women overcome barriers to lifestyle change, align interventions with their recovery trajectory and caregiving demands, and ultimately support longer-term cardiovascular health.

### 4.1. Strengths and Limitations

A key strength of this study is its integration within the BP^2^ randomised controlled trial, which, to our knowledge, is the largest RCT to date focused on structured follow-up and lifestyle behaviour change in women following HDP. This qualitative study’s strengths include the depth of insight gained through thematic analysis of women’s HDP experiences across different intervention arms and life contexts. The diagnosis of HDP was made using the ISSHP definition [2] and was consistent across all study sites. However, these findings should be interpreted considering several limitations. Although the interview sample was broadly representative of the overall BP^2^ cohort, participants were more likely to be first-time mothers, who may have greater difficulty adjusting to parenthood or greater capacity to engage in lifestyle behaviour change compared to women with multiple children. Although the BP^2^ study included women from many different socioeconomic backgrounds, this sub-study primarily reflects the perspectives of the study sample and may not fully capture the experiences of women from more socioeconomically disadvantaged backgrounds or culturally diverse contexts. Women in these groups may face additional or distinct barriers to lifestyle change, including limited access to healthcare resources, financial constraints, or culturally specific caregiving expectations. Furthermore, women enrolled in the broader BP^2^ study may not fully reflect the general population of those who have experienced HDP. The interviews were conducted at a single time point (10–12 months postpartum), which may not capture the dynamic nature of women’s readiness for behaviour change over time. While this timing allowed participants to provide reflective accounts of their postpartum experiences, it inherently limits insight into how perceptions, priorities, and lifestyle behaviours may evolve beyond the first year. Finally, it is important to acknowledge the potential influence of the COVID-19 pandemic on interviewees’ reported lifestyle behaviours and perceived barriers. Some interviews were conducted during periods of lockdown in Sydney, which limited access to exercise facilities, social support, and health services, and increased caregiving demands and stress. These contextual factors are likely to have shaped women’s experiences and should be considered when interpreting the findings.

### 4.2. Future Directions

Further research should explore how to time and tailor interventions to maximise uptake and effectiveness, using appropriate behaviour change theory and potentially through staggered or modular approaches. Partner and family involvement, integration with digital health tools, and culturally responsive models of care may also enhance engagement. Long-term follow-up is needed to determine whether increased awareness and behavioural change translate into improved cardiovascular outcomes for women with a history of HDP.

## 5. Conclusions

Drawing on women’s reported experiences, this qualitative study highlights the postpartum experience following HDP, including physical recovery, emotional processing, and intensive infant care, particularly after preterm birth. Following HDP, it often takes time before women are ready to prioritise their own health and engage in lifestyle change. These findings highlight the perceived importance of flexible, accessible interventions that accommodate individual recovery, caregiving demands, and available support networks. While not establishing causal effects, the insights generated from women’s experiences suggest that tailoring postpartum support in this way may better align with women’s needs and potentially contribute to reducing women’s long-term CVD risk, addressing one of the key consequences of HDP.

## Data Availability

The data presented in this study are available on request from the corresponding author due to ethical reasons. The minimum dataset has been provided in the Coding Frame.

## Abbreviations

The following abbreviations are used in this manuscript:

HDP	Hypertension disorders of pregnancy
CVD	Cardiovascular disease
CH	Chronic hypertension
GH	Gestational hypertension
RCT	Randomised control trial
BP^2^	Blood Pressure Postpartum Study
NSW	New South Wales
GHS	Get Healthy Service
ANZCTR	Australian and New Zealand Clinical Trials Register
AUD	Australian dollar
ISSHP	International Society for the Study of Hypertension in Pregnancy

## Appendix A

**Table A1:** Interviewee characteristics compared with BP^2^ cohort.

	Interviewees N = 34			BP^2^ Cohort N = 484		
	n	%	Mean	N	%	Mean
Age (years)			33 (5.3)			34 (5.2)
Country of birth						
Australia	26	76		285	59	
Not Australia	8	24		199	41	
Highest level of education						
Secondary school	4	12		46	10	
Trade certificate/diploma	9	26		108	22	
University degree	21	62		326	67	
No data available	-	-		4	1	
Parity						
Primipara	29	85		326	67	
Multipara	5	15		158	33	
Plurality						
Singleton	32	94		468	97	
Twins	2	6		16	3	
HDP						
Chronic hypertension	2	6		75	16	
Gestational hypertension	9	26		114	23	
Preeclampsia	20	59		268	55	
Chronic Hypertension + Preeclampsia	3	9		27	6	
Gestation at birth (weeks)			37 (2.6)			37 (3.1)
≥37^+0^ weeks	26	76		349	72	
34^+0^–36^+6^ weeks	5	15		76	16	
<34^+0^ weeks	3	9		59	12	

## Appendix B

Interview Guide Mapped to the Social Ecological Model (SEM)

Group 1—Optimised Usual Care. Info pack (Get Healthy Service and Heart Foundation).

Thank you very much for agreeing to this interview. As you may know we are interviewing women who are taking part in the Blood Pressure Postpartum study. We are talking to women who have had high blood pressure in pregnancy to find out what things make it harder and easier to keep healthy. We are also interested in your feedback about being in the Blood Pressure Postpartum study.

I would just like to confirm that you are happy for me to record this interview?

I realise that you have filled in various questionnaires, but I don’t have access to that information. I know that you had a little boy/girl last year, but I don’t know their name. I’d like to use a name in the interview, so you can tell me their name if you like or use a pseudonym. I realise you may have to leave the interview to attend to him/her. That’s fine—I can always call you back later. And please remember you can skip over any questions you don’t feel comfortable to answer.

**Table A2:** Group 1 interview questions.

First of all, I’d like to ask you about your diet and eating habits….	SEM Domain
Have your eating habits changed since you had your baby? How has your diet changed since having (baby name)? How does that compare with when you were pregnant? How does that compare with before you were expecting (baby name)? [If mention changes in diet] Have these changes had an impact on your everyday life? How? [If mention changes in diet] How have these changes affected your family or the people you live with? Have you faced any challenges in eating a healthy diet? [You already mentioned xxxx, is there anything else that makes it hard?] Has anything helped you in eating a healthy diet? Have you received support from your family or friends? Do you feel that eating healthy food is important to your friends and family?	Individual Interpersonal Interpersonal
Now I have some questions about physical activity….	
Have your exercise habits changed since you had your baby? How has your level of physical activity changed since having (baby name)? How does that compare with when you were pregnant? How does that compare with before you were expecting (baby name)? [If mention changes in PA] Have these changes had an impact on your everyday life? How? [If mention changes in PA] How have these changes affected your family or the people you live with? Have you faced any challenges in being physically active? [You already mentioned xxxx, are there other factors that make it hard?] Has anything helped you in keeping active? Have you received support from your family or friends? Do you feel that being physically active is important to your friends and family?	Individual Interpersonal Interpersonal
Looking now at healthy lifestyle in general….	
J. Have you experienced any challenges to other aspects of your health since you had (baby name)? Like smoking or drinking? K. If so, did this involve your family or friends?	Individual Interpersonal
L. How motivated are you to eat healthy food and participate in physical activity? M. Has participating in the BP^2^ program made a difference to your motivation?	Individual Organisation
N. Do you feel confident that you can maintain the lifestyle changes you’ve made (if any and if positive)? O. Can you think of anything that might make it easier (or harder) for you to maintain these changes? P. Have you found any groups or services in your local area that have helped you adopt a healthy lifestyle and maintain a healthy weight? Q. What do you think organisations could do to help mums like you to eat well and be physically active? Like workplaces or local councils? R. Could governments do anything?	Individual Community Policy
Now I’m going to ask about your contact with health professionals	
1.1 Have you discussed cardio-vascular health with your GP or practice nurse? Have you discussed diet and PA? Can you tell me a bit about the information they gave you? Was there anything that was particularly helpful?	Community
1.2 Do you recall receiving an information pack from the BP^2^ team about keeping healthy after having high blood pressure in pregnancy? You would have received that after you signed up for the blood pressure research, when (baby name) was about six months. If YES, was it helpful?	Organisation
1.3 Do you remember receiving a flyer about the NSW Get Healthy Service? 1.4 Did you contact the GHS? If NO—stop interview. No further questions. If YES 1.5 What made you decide to join GHS? (PROMPT: goals?) 1.6. How many calls do you recall with your health coach? Was it the same coach each time? 1.7 Did the GHS calls suit you? (PROMPT: number of calls, timing of calls, convenience, fitting with parenting routines, cultural appropriateness). Did it suit your family? 1.8 What did you like about the GHS? What did you dislike? (PROMPT: Talking on the phone about your health, feelings about health coach—gender?) 1.9 Has being part of the GHS helped you? How? 1.10 If there was anything about GHS that you would change, what would that be?	Organisation

That is the end of my questions… unless there is anything else you would like to add about what we’ve been talking about. Thank you so much for giving me the time for this interview. We will send your gift voucher in the next week or so.

Group 2—Brief Education intervention. Info pack (Get Healthy Service and Heart Foundation) + visit to follow up clinic at 6 months—discussion with doctor and dietitian.

Thank you very much for agreeing to this interview. As you may know we are interviewing women who are taking part in the Blood Pressure Postpartum study. We are talking to women who have had high blood pressure in pregnancy to find out what things make it harder and easier to keep healthy. We are also interested in your feedback about being in the Blood Pressure Postpartum study.

I would just like to confirm that you are happy for me to record this interview?

I realise that you have filled in various questionnaires, but I don’t have access to that information. I know that you had a little boy/girl last year, but I don’t know their name. I’d like to use a name in the interview, so you can tell me their name if you like or use a pseudonym. I realise you may have to leave the interview to attend to him/her. That’s fine—I can always call you back later. And please remember you can skip over any questions you don’t feel comfortable to answer.

**Table A3:** Group 2 interview questions.

First of all, I’d like to ask you about your diet and eating habits….	SEM Domain
J. Have your eating habits changed since you had your baby? K. How has your diet changed since having (baby name)? L. How does that compare with when you were pregnant? M. How does that compare with before you were expecting (baby name)? N. [If mention changes in diet] Have these changes had an impact on your everyday life? How? O. [If mention changes in diet] How have these changes affected your family or the people you live with? P. Have you faced any challenges in eating a healthy diet? [You already mentioned xxxx, is there anything else that makes it hard?] Q. Has anything helped you in eating a healthy diet? Have you received support from your family or friends? R. Do you feel that eating healthy food is important to your friends and family?	Individual Interpersonal Interpersonal
Now I have some questions about physical activity….	
S. Have your exercise habits changed since you had your baby? T. How has your level of physical activity changed since having (baby name)? U. How does that compare with when you were pregnant? V. How does that compare with before you were expecting (baby name)? W. [If mention changes in PA] Have these changes had an impact on your everyday life? How? X. [If mention changes in PA] How have these changes affected your family or the people you live with? Y. Have you faced any challenges in being physically active? [You already mentioned xxxx, are there other factors that make it hard?] Z. Has anything helped you in keeping active? Have you received support from your family or friends? AA. Do you feel that being physically active is important to your friends and family?	Individual Interpersonal Interpersonal
Looking now at healthy lifestyle in general….	
BB. Have you experienced any challenges to other aspects of your health since you had (baby name)? Like smoking or drinking? CC. If so, did this involve your family or friends?	Individual Interpersonal
DD. How motivated are you to eat healthy food and participate in physical activity? EE. Has participating in the BP^2^ program made a difference to your motivation?	Individual Organisation
FF. Do you feel confident that you can maintain the lifestyle changes you’ve made (if any and if positive)? GG. Can you think of anything that might make it easier (or harder) for you to maintain these changes? HH. Have you found any groups or services in your local area that have helped you adopt a healthy lifestyle and maintain a healthy weight? II. What do you think organisations could do to help mums like you to eat well and be physically active? Like workplaces or local councils? JJ. Could governments do anything?	Individual Community Organisation Policy
Now I’m going to ask about your contact with health professionals	
2.1 Have you discussed cardio-vascular health with your GP or practice nurse? Have you discussed diet and PA? Can you tell me a bit about the information you received? Was there anything that was particularly helpful?	Community
2.2 Do you recall receiving an information pack from the BP^2^ team about keeping healthy after having high blood pressure in pregnancy? You would have received that after you signed up for the blood pressure research, when (baby name) was about six months. If YES, 2.3 Was it helpful?	Organisation
2.4 Do you remember receiving a flyer about the NSW Get Healthy Service? If NO—go to Q2.12 2.5 Did you contact the GHS? If NO—Go to Q2.12 If YES 2.6 what made you decide to join GHS? (PROMPT: goals?) 2.7 How many calls do you recall with your health coach? Was it the same coach each time? 2.8 Did the GHS calls suit you? (PROMPT: number of calls, timing of calls, convenience, fitting with parenting routines, cultural appropriateness). Did it suit your family? 2.9 What did you like about the GHS? What did you dislike? (PROMPT: Talking on the phone about your health, feelings about health coach—gender?) 2.10 Has being part of the GHS helped you? How? 2.11 If there was anything about GHS that you would change, what would that be?	Organisation
2.12 Did you visit the hospital clinic as part of the BP^2^ study, when your baby was about 6 months old? If YES Can you tell me a bit about the visit? Was there anything you particularly remember about the visit? Or any helpful information or advice you received?	Organisation

That is the end of my questions… unless there is anything else you would like to add about what we’ve been talking about. Thank you so much for giving me the time for this interview. We will send your gift voucher in the next week or so.

Group 3—Extended lifestyle intervention. Info pack (Get Healthy Service and Heart Foundation) + visit to follow up clinic at 6 months—discussion with doctor and dietitian + lifestyle behaviour coaching through Get Healthy Service.

Thank you very much for agreeing to this interview. As you may know we are interviewing women who are taking part in the Blood Pressure Postpartum study. We are talking to women who have had high blood pressure in pregnancy to find out what things make it harder and easier to keep healthy. We are also interested in your feedback about being in the Blood Pressure Postpartum study.

I would just like to confirm that you are happy for me to record this interview?

I realise that you have filled in various questionnaires, but I don’t have access to that information. I know that you had a little boy/girl last year, but I don’t know their name. I’d like to use a name in the interview, so you can tell me their name if you like or use a pseudonym. I realise you may have to leave the interview to attend to him/her. That’s fine—I can always call you back later. And please remember you can skip over any questions you don’t feel comfortable to answer.

**Table A4:** Group 3 interview questions.

First of all, I’d like to ask you about your diet and eating habits….	SEM Domain
S. Have your eating habits changed since you had your baby? T. How has your diet changed since having (baby name)? U. How does that compare with when you were pregnant? V. How does that compare with before you were expecting (baby name)? W. [If mention changes in diet] Have these changes had an impact on your everyday life? How? X. [If mention changes in diet] How have these changes affected your family or the people you live with? Y. Have you faced any challenges in eating a healthy diet? [You already mentioned xxxx, is there anything else that makes it hard?] Z. Has anything helped you in eating a healthy diet? Have you received support from your family or friends? AA. Do you feel that eating healthy food is important to your friends and family?	Individual Interpersonal Interpersonal
Now I have some questions about physical activity….	
KK. Have your exercise habits changed since you had your baby? LL. How has your level of physical activity changed since having (baby name)? MM. How does that compare with when you were pregnant? NN. How does that compare with before you were expecting (baby name)? OO. [If mention changes in PA] Have these changes had an impact on your everyday life? How? PP. [If mention changes in PA] How have these changes affected your family or the people you live with? QQ. Have you faced any challenges in being physically active? [You already mentioned xxxx, are there other factors that make it hard?] RR. Has anything helped you in keeping active? Have you received support from your family or friends? SS. Do you feel that being physically active is important to your friends and family?	Individual Interpersonal Interpersonal
Looking now at healthy lifestyle in general….	
TT. Have you experienced any challenges to other aspects of your health since you had (baby name)? Like smoking or drinking? UU. If so, did this involve your family or friends?	Individual Interpersonal
VV. How motivated are you to eat healthy food and participate in physical activity? WW. Has participating in the BP^2^ program made a difference to your motivation?	Individual Organisation
XX. Do you feel confident that you can maintain the lifestyle changes you’ve made (if any and if positive)? YY. Can you think of anything that might make it easier (or harder) for you to maintain these changes? ZZ. Have you found any groups or services in your local area that have helped you adopt a healthy lifestyle and maintain a healthy weight? AAA. What do you think organisations could do to help mums like you to eat well and be physically active? Like workplaces or local councils? BBB. Could governments do anything?	Individual Community Policy
Now I’m going to ask about your contact with health professionals	
3.1 Have you discussed cardio-vascular health with your GP or practice nurse? Have you discussed diet and PA? Can you tell me a bit about the information you received? Was there anything that was particularly helpful?	Community
3.2 Do you recall receiving an information pack from the BP^2^ team about keeping healthy after having high blood pressure in pregnancy? You would have received that after you signed up for the blood pressure research, when (baby name) was about six months. If YES, was it helpful?	Organisation
3.3 Did you visit the hospital clinic as part of the BP^2^ study, when your baby was about 6 months old? Can you tell me a bit about the visit? Was there anything you particularly remember about the visit? Or any helpful information or advice you received?	Organisation
3.5 Have you been in touch with the Get Healthy Service? If NO, why is that? AND STOP Interview. No further questions If YES. 3.6 When you were enrolled in the GHS, what did you hope to get out of the program? (PROMPT: goals?) 3.7 How many calls do you recall with your health coach? Was it the same coach each time? 3.8 Did the GHS calls suit you? (PROMPT: number of calls, timing of calls, convenience, fitting with parenting routines, cultural appropriateness). Did it suit your family? 3.9 What did you like about the GHS? What did you dislike? (PROMPT: Talking on the phone about your health, feelings about health coach—gender?) 3.10 Has being part of the GHS helped you? How? 3.11 If there was anything about GHS that you would change, what would that be?	Organisation

That is the end of my questions… unless there is anything else you wish to add about what we’ve been talking about. Thank you so much for giving me the time for this interview. We will send your gift voucher in the next week or so.
